# CT-Guided Percutaneous Cryoablation of Small Renal Masses: General Anesthesia Versus Conscious Sedation with Dexmedetomidine

**DOI:** 10.1007/s00270-025-04079-7

**Published:** 2025-06-06

**Authors:** Theresa Junker, Nina Stage, Mie Gaedt Thorlund, Benjamin Schnack Brandt Rasmussen, Bo Redder Mussmann, Tommy Kjærgaard Nielsen, Jakob Kjersgaard Johansen, Ole Graumann

**Affiliations:** 1 Present Address: Research and Innovation Unit of Radiology - UNIFY, University of Southern Denmark, Odense, Denmark; 2 Department of Urology, Odense University Hospital (OUH), Odense, Denmark; 3 Department of Radiology, OUH, Odense, Denmark; 4 Department of Urology, Aalborg University Hospital, Aalborg, Denmark; 5 Department of Anesthesiology and Intensive Care, Neuroanesthesia (OUH), Odense, Denmark; 6 Department of Radiology, Aarhus University Hospital, Aarhus, Denmark; 7 Department of Clinical Medicine, Aarhus University, Aarhus, Denmark

**Keywords:** Renal tumor, RCC, Cryoablation, General anesthesia versus conscious sedation

## Abstract

**Purpose:**

General anesthesia (GA) is often used during CT-guided percutaneous cryoablation (PCA) of renal tumors. This retrospective study compared GA to conscious sedation (CS), with dexmedetomidine and remifentanil, regarding theater time and hospital stay during PCA of renal tumors.

**Materials and Methods:**

This retrospective study reviewed 350 patients treated with PCA between January 1, 2015, and December 31, 2019. Associations were analyzed between the type of anesthesia and theater time, hospital stay, complications, and clinical outcomes.

**Results:**

The cohort consisted of 148 patients who received PCA in GA (mean age 64.7 ± 13.4 years; 99 men) and 202 patients who received PCA in CS (mean age 66.7 ± 10.7 years; 142 men). Patients in the GA group had significantly longer theater times (mean 2.6 h ± 0.48) compared to their CS counterparts (mean 2.1 h ± 0.46; *p* < 0.001). Furthermore, the median length of hospital stay was significantly longer in the GA group (8.5 vs. 5.5 h, *p* < 0.001). In addition, patients in the GA group had significantly lower levels of consciousness in the recovery room compared to the CS group (*p* = 0.047). There were no differences between the groups regarding complications, graded on the CIRSE classification system (*p* = 0.463), or rate of incomplete ablation at three-month follow-up (*p* = 0.229).

**Conclusion:**

Patients who undergo PCA of renal tumors under CS have significantly shorter theater time and length of hospital stay compared to patients who undergo PCA in GA, with no impact on complications or technical failure rate.

**Level of Evidence:**

3, a retrospective cohort study.

## Introduction

In 2018, there were an estimated 136.5 new cases of renal cell carcinoma (RCC) per 100,000 inhabitants in Europe [1, 2]. Traditionally, RCC has been treated surgically with partial or radical nephrectomy [3]. Over the last two decades, considerable advances have been made in minimally invasive, nephron-sparing procedures, such as computed tomography (CT) guided percutaneous cryoablation (PCA) [4]. The safety and efficacy of PCA have resulted in an increasing number of PCAs being performed in curative treatment of RCC stage T1a [5–7].

General anesthesia (GA) with endotracheal intubation has been the standard during PCA of renal tumors for many interventional radiologists. GA is used for many types of procedures but is associated with the risk of anesthesia-related complications, especially in patients who are elderly or have multiple comorbidities [8]. However, PCA has previously been described as nearly painless, eliminating the need for GA or deep sedation [9]. European guidelines suggest that PCA of renal tumors can be used for frail and/or comorbid patients with small renal masses [3, 10], which is supported by Morkos et al., finding PCA to be associated with prolonged overall survival compared to surgical alternatives, especially in patients with comorbidities [11]. However, advanced age and comorbidities present a risk of complications, causing physicians to be cautious about PCA due to the risks associated with GA.

PCA performed under conscious sedation (CS) has been promoted during the last two decades [5, 7–9, 12]. Studies have previously used benzodiazepines, known central cerebral inhibitors, for CS during PCA with promising results [13]. Dexmedetomidine is an α_2_-adrenoceptor agonist and, thus, is not a central cerebral inhibitor [13]. It is a common choice of sedative in intensive care settings. It has also been used during non-invasive or more minor procedures, where deep sedation or GA is considered excessive [13–15]. The use of Dexmedetomidine in CS has not previously been investigated as a choice of sedation during PCA; however, its use during such procedures could prove advantageous. A combination of dexmedetomidine and remifentanil, an ultra-short-acting opioid, may be vital in ensuring patient comfort during PCA due to the combined sedative, analgesic, and anxiolytic effects of the two medications [13, 14, 16, 17].

This retrospective study compared GA to CS with dexmedetomidine during PCA of renal tumors regarding theater time, length of hospital stay, and clinical outcomes.

## Materials and Methods

This retrospective study was reported according to the Strengthening and Reporting of Observational Studies in Epidemiology guideline (STROBE) [18].

### Ethical Considerations

The present study was conducted at Odense University Hospital (OUH) in Denmark, with approval from the institutional review board and the Danish Data Protection Agency (no. 20/290).

### Setting and Patient Selection

Patients treated with PCA of renal tumors between January 1, 2015, and December 31, 2019, were considered eligible for inclusion. Inclusion criteria were PCA of suspected or biopsy-proven renal masses and completion of PCA procedure. Exclusion criteria were procedures for non-renal masses. The treatment decisions were based on a multidisciplinary team conference, and all patients underwent preprocedural examination by a urologist and an anesthesiologist. Until June 2017, GA was the only type of anesthesia used during PCA of renal tumors. After that date, CS with a combination of dexmedetomidine and remifentanil was implemented as the standard method. Patients who did not want to undergo PCA under CS or were deemed unable to cooperate during the procedure (i.e., patients with cognitive dysfunctions or language barriers) were treated under GA.

### Data Collection and Variables of Interest

Information was collected from the Picture Archiving and Communications System (PACS) and electronic medical records. Recorded patient variables included demographics (gender, height, weight, and American Society of Anesthesiologists score), tumor characteristics (size, RENAL score, biopsy histopathology), and procedure characteristics (type of anesthesia).

### Outcome Measures

The primary outcome variables were theater time and length of hospital stay for patients undergoing PCA with GA compared to CS. Theater time was defined as the collected time spent in the CT room. The length of hospital stay was defined as the time between arrival at the CT room and discharge from the hospital.

Secondary outcomes included: evaluation of pain on a numeric rating scale (NRS) from 0 to 10, where 0 was no pain, and 10 was maximum pain; nausea and level of consciousness (on scales from 0 to 3, with 0 being no nausea and 3 being severe nausea, and 0 being no affection of consciousness and 3 being unconsciousness non-responsive to verbal or physical stimulation). Pain, nausea, and level of consciousness were evaluated during the stay in the recovery room (1–2 h after the end of the PCA procedure). Complications were recorded during hospitalization and classified by the Cardiovascular and Interventional Radiological Society of Europe (CIRSE) Classification System for Complications [19]. Finally, readmission within 90 days and incomplete ablation were recorded. Incomplete ablation was defined as nodular enhancement of the target lesion on contrast-enhanced CT at the three-month follow-up.

### Procedure

Preoperatively, patients were asked to omit anticoagulants, oral antidiabetics, and most antihypertensive medications from their daily intake. Two hours before the procedure, patients were administered paracetamol and dexamethasone, which were practiced for patients in the GA and CS groups. Patients with diabetes were treated under an insulin-based regime.

Patients were treated either under GA with propofol and remifentanil (Ultiva, Aspen Nordic) and intubated or under CS with a combination of dexmedetomidine (Dexdor, Orion Pharma) and remifentanil (Ultiva, Aspen Nordic) with lidocaine and Marcaine as local anesthesia. The CS patients could position themselves on the CT table without assistance, while GA patients were manually repositioned upon intubation.

PCA was performed by three senior radiologists with 3, 6, and 15 years of experience with interventional radiology under CT guidance (Siemens SOMATOM Flash, Erlangen, Germany), respectively. Hydro dissection with 2% iodine-based saline solution was applied when deemed necessary. Cryoprobes [14- or 17-gauge] were percutaneously applied to the tumor. The procedure was performed with an argon-based system (Visual Ice, Boston Scientific, MN, USA, and ICEfx, Boston Scientific, MN, USA). The system induces two 10-min freezes separated by an 8-min thaw. During freezing, CT images were obtained to ensure that the ice ball extended the tumor by a minimum of 5 mm for sufficient treatment and to observe any complications [20].

Patients in the GA group were extubated and assisted to their beds using transfer boards. A few minutes after the procedure, patients in the CS group could walk to their beds independently. All patients were then transferred to the recovery room. From there, they were transported to the Department of Urology and kept under observation until discharged.

### Statistical Analyses

Differences between continuous variables were assessed with the Student’s t-test for normally distributed data or the Mann–Whitney U test for data that were not. Categorical variables were compared using the χ^2^-test, except for the biopsy histopathology data, incomplete ablation, and readmission, where Fisher’s exact test was applied. *p*-values were significant at < 0.05. Statistical analyses were performed using STATA/IC 16 (StataCorp LP, College Station, TX, USA).

## Results

### Study Participants

**Fig. 1:**
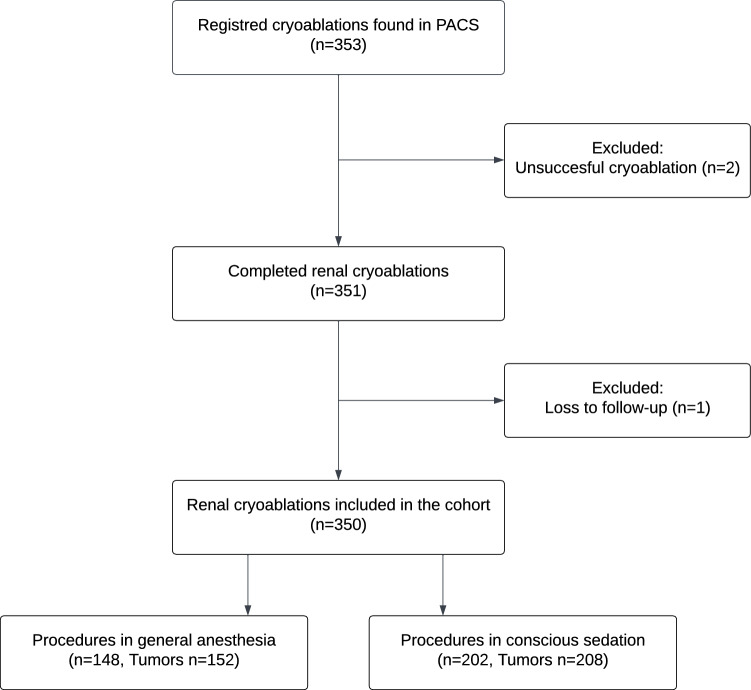
Flow diagram of patient inclusion. PACS: Picture Archiving and Communications System

In total, 353 PCA procedures of renal tumors were identified from January 1, 2015, to December 31, 2019. Two patients were excluded due to ineffective hydrodissection and, therefore, inability to complete PCA, and one patient was excluded due to loss of follow-up. Thus, the total number of PCA procedures included in this study was 350 on 331 patients with 360 tumors. A total of 148 patients (mean age, 64.7 ± 13.4 years; 99 men) underwent GA, and 202 (mean age, 66.7 ± 10.7 years; 142 men) underwent CS (Fig. 1). In 19 procedures, PCA was performed on residual tumor tissue either due to incomplete treatment or recurrence.

Table 1 summarizes the characteristics of patients and tumors. No significant differences were found between the GA and CS groups concerning gender, age, body mass index, or ASA score. In addition, no significant differences were found in the tumor characteristics: size, RENAL score, and biopsy pathology. Missing data on biopsy pathology resulted from either no biopsy being performed before PCA or a lack of included pathology results.

**Table 1:** Baseline patient and tumor characteristics

Characteristics	GA (n = 148)	CS (n = 202)	*p* value
Age (y)^a^	64.7 ± 13.4	66.7 ± 10.7	0.236
No. of men, n (%)	99 (66.9)	142 (70.3)	0.497
BMI (kg/m^2^)^a^	28.1 ± 6.3	28.1 ± 5.3	0.372
ASA score, n (%)			0.606
1	15 (10.1)	15 (7.4)	
2	76 (51.4)	108 (53.5)	
3	56 (37.8)	74 (36.6)	
4	1 (.7)	5 (2.5)	
Tumor size (mm)^a^	27.6 ± 9.7	28.2 ± 11.2	0.693
RENAL score^a^	7 ± 1.8	7.02 ± 1.8	0.754
Biopsy pathology, n (%)			0.128^b^
No. of observations = 360	Observed no. = 152	Observed no. = 208	
Clear cell RCC	98 (63.1)	114 (54.8)	
Papillary RCC	27 (17.6)	55 (26.4)	
Chromophobe RCC	7 (4.6)	13 (6.3)	
Metastasis	3 (2)	1 (.5)	
Mixed clear cell/pap RCC	1 (.7)	1 (.5)	
Unclassified RCC	6 (3.9)	5 (2.4)	
Angiomyolipoma	2 (1.3)	10 (5.3)	
Oncocytoma	1 (.7)	1 (.5)	
Inconclusive	2 (1.3)	1 (.5)	
Adenocarcinoma	0 (0)	1 (.5)	
Missing	5 (3.9)	6 (2.4)	
Maglignant/non-malignant^*b^			0.166
Malignant	142 (98.0)	190 (95.0)	
Non-malignant	3 (2.0)	11 (5.5)	

Unless otherwise specified, data is numbers of cryoablated patients, with percentage in parentheses

ASA = American Society of Anesthesiologists, BMI = Body Mass Index, CS = Conscious Sedation, GA = General Anesthesia, pap = papillary, RCC = Renal Cell Carcinoma, RENAL = Radius, Exophytic/Endophytic properties, Nearness of the tumor to the collecting system or sinus, Anterior/posterior, and Location

^a^Mean ± standard deviations

^b^Fischer’s exact test was applied due to low representation in some tumor groups

*Missing or inconclusive biopsy not included n = 14

75% (n = 261) of the cohort were treated after conscious sedation was implemented in June 2017. Table 2 shows the distribution of conscious sedation and general anesthesia use from June 1, 2017, to December 31, 2019.

**Table 2:** Distribution of patients treated with conscious sedation or general anesthesia from June 1, 2017 to December 31, 2019

Time period	GA (n = 59)	CS (n = 202)
2017*	42 (59)	29 (41)
2018	9 (9)	90 (91)
2019	8 (9)	83 (91)

Data is numbers of cryoablated patients, with percentages in parentheses

CS = Conscious Sedation, GA = General Anesthesia

*June 1 to December 31, 2017

### Primary Outcomes

Table 3 summarizes the outcomes of the procedures. Mean theater time was 2.6 h ± 0.48 in the GA group and 2.1 h ± 0.46 in the CS group (*p* < 0.001). For hospital stay, the median time in the GA group was 8.5 h (IQR 18.98) and 5.5 h (IQR 2.23) in the CS group (*p* < 0.001).

**Table 3:** Primary and secondary outcomes for patients treated with PCA under GA or CS during hospitalization and follow-up

Variables	GA (n = 148)	CS (n = 202)	*p*-value
Theater time (h)^a^	2.6 ± 0.48	2.1 ± 0.46	< 0.001
Hospital stay (h)^b^	8.5 (18.98)	5.5 (2.23)	< 0.001
Recovery room^a^*			
NRS-score	0.728 ± 1.28	0.792 ± 1.348	0.875
Nausea-score	0.082 ± .321	0.089 ± .376	0.908
Level of consciousness-score	0.272 ± .518	0.158 ± .366	0.047
Follow-up, n (%)			
Incomplete ablation**	8 (5.6)	6 (3.0)	0.229
Readmission**	4 (2.8)	8 (4.0)	0.523

Unless otherwise specified, data is numbers of cryoablated patients, with percentages of patients in parentheses. One patient was admitted to the Intensive Care Unit due to cardiac arrest at the end of the procedure and does not have any registered recovery room scores

CS = Conscious Sedation, GA = General Anesthesia, NRS = Numeric Rating Scale

^a^Mean ± standard deviations

^b^Median time with IQR

*GA n = 147

**GA n = 144

### Secondary Outcomes

There were no differences in pain and nausea scores during the stay in the recovery room between the groups. However, there was a significant difference regarding the level of consciousness (mean 0.272 in the GA group vs 0.158 in the CS group; *p* = 0.047).

The rate of incomplete ablation at the three-month follow-up was similar between the groups, 5.6% in the GA group compared to 3% in the CS group (*p* = 0.229). In addition, no differences were found in readmission rates between the groups, 2.8% in the GA group versus 4% in the CS group (*p* = 0.523).

Finally, twenty patients across both groups (6.8% in the GA group versus 5% in the CS group) experienced complications during hospitalization. The most frequently reported complication was pneumothorax (n = 5). Two patients with pneumothorax in the CS group needed drainage beyond the procedure, grade 3a on the CIRSE classification, while the rest were either resolved during the procedure or followed up with an X-ray, grade 1a or grade 2 on CIRSE. Four (2.7%) in the GA group and one (0.5%) in the CS group experienced urine retention, grade 3a on CIRSE, which was solved by bladder catheterization. Finally, one patient in the GA group had a cardiac arrest following the removal of the endotracheal tube (CIRSE grade 3a). Table 4 summarizes complications during hospitalization, graded on the CIRSE classification system for complications.

**Table 4:** Complications during hospitalization graded on the CIRSE classification system for complications

Grades	Complications	GA	CS	*p* value
1a	Pneumothorax (periprocedural drainage), freeze injury of the skin from cryoprobes	2 (1.4)	3 (1.5)	*p* = 0.463
2	Pneumothorax (follow-up x-ray)	1 (0.7)	1 (0.5)	
3a	Urine retention (catheterized), painful hematoma (blood transfusion), pneumothorax with lasting drainage, renal artery hemorrhage (embolized), cardiac arrest (revived)	7 (4.7)	6 (3.0)	
Total		10 (6.8)	10 (5.0)	

Data is number of patients with percentages in parentheses. Complications are written with their respective treatments in parentheses

## Discussion

This retrospective study investigated the differences among patients undergoing PCA of renal tumors who received either GA or CS during the procedure. Results showed that patients undergoing PCA in CS had a significantly shorter theater time (*p* < 0.001) and shorter hospital stays (*p* < 0.001) compared to patients undergoing PCA in GA. Furthermore, no differences in complications under hospitalization or incomplete ablations during follow-up were found. However, the level of consciousness during the stay in the recovery room was significantly lower for patients in the GA group (*p* = 0.047).

The differences in theater time between the groups reported in the present study can be explained by GA being more time-consuming during PCA since it requires endotracheal intubation. Furthermore, patients must be repositioned while anesthetized, intubated, and connected to the monitoring equipment [21]. The benefits of GA may, however, outweigh the time-saving capabilities of CS for some interventional radiologists [22–24]. Thus, the advantages of GA mainly encompass regulating respiration, which may facilitate cryoprobe placement, and management of respiratory problems if the patient is in a prone position. However, all the procedures in the present study were performed with the patient in a side position. Another advantage of having the patient unconscious could be in cases where a communication barrier exists between the patient and the interventional radiologist. Furthermore, patients with illnesses affecting cooperation during the procedure (such as cognitive dysfunctions) may benefit from GA during PCA. In the present study, GA continued to be an option in cases such as these or cases of patient preferences. After implementing CS in June 2017, 59% of patients treated received GA, mainly due to organization issues. However, a decrease in the use of GA to 9% was observed in 2018 and 2019, where the main reason for GA was cognitive dysfunctions and unique patient preferences.

CS with dexmedetomidine has been associated with great satisfaction among patients and physicians in interventions, such as oral surgery, cataract surgery, bronchoscopy, and transurethral prostate resection [13]. To our knowledge, no previous articles have described dexmedetomidine (alone or in combination with remifentanil) as a choice of sedation when performing PCA of renal tumors. However, benzodiazepines are widely applied for the same purpose [9, 25, 26]. A reported adverse event with benzodiazepines is that they can create paradoxical reactions (anxiety, restlessness, agitation, aggression) in patients during the procedure [13, 16, 27]. Compared to benzodiazepines, dexmedetomidine, combined with the use of remifentanil, provides a comfortable procedure for the patient [13, 16, 28, 29]. Okhunov et al. compared CS with benzodiazepines to GA regarding procedure time and hospital stay [8]. They reported that patients who underwent PCA under sedation with benzodiazepine spent less time hospitalized: approximately 24 h in the CS group and almost two days in the GA group. In the present study, we report a shorter median hospitalization in both groups than Okhunov et al. [8]. However, the results in the present study correspond to the results by Okhunov et al.; CS seems more time-efficient than GA when patients undergo PCA of renal tumors [8]. This saves time for both patients and healthcare professionals. For instance, our institution’s daily capacity increased from 2–3 procedures per day to a minimum of 3 procedures per day when CS became the standard method. Furthermore, CS offers a quick recovery from anesthesia and an earlier hospital discharge [8].

The present study found no differences in complications during hospitalization between the groups, which indicates that CS is a safe anesthetic during PCA of renal tumors for vulnerable, comorbid patients who may face complications from GA or have even been denied cancer treatment because of them [8, 15, 25, 30]. The results do not encompass complications beyond discharge. However, it seems unlikely that CS should be the cause of complications beyond discharge.

A limitation of the present study is that the included patients were not randomly allocated to GA or CS, which makes it prone to selection bias. Furthermore, the retrospective study design introduces limitations, such as the risk of incomplete data capture or potential confounding variables.

Finally, CS became the preferred anesthetic during renal tumor PCA after the interventional radiologists in this study spent years gaining experience with it under GA. This might have resulted in an experience bias, given that increased experience could have influenced the speed of the procedure relative to the choice of anesthetic. However, the procedures were performed by three different interventional radiologists with varying experience levels, which could also contribute to variability.

In conclusion, patients who underwent PCA of renal tumors under CS with dexmedetomidine had significantly shorter theater time and length of hospital stay compared to patients who underwent PCA in GA, with no impact on complications or oncological outcomes.
